# Birth growth curves of neonates in high-altitude areas: A cross-sectional study

**DOI:** 10.3389/fped.2022.1028637

**Published:** 2023-01-10

**Authors:** Bo Wang, Yan-Li Yao, Jing Kang, Cun-Gui Li, Guo-Fei Zhang, Zhang-Bin Yu

**Affiliations:** ^1^ Department of Pediatrics, The Affiliated Suqian First People's Hospital of Nanjing Medical University, Suqian, China; ^2^ Department of Neonatology, Qinghai Red Cross Hospital, Xining, China; ^3^ Department of Neonatology, Shenzhen People's Hospital, Shenzhen, China

**Keywords:** birth weight, Chinese standard, growth curve, high altitude, INTERGROWTH-21st project, neonate

## Abstract

**Background:**

Since the current commonly used birth growth curves are unsuitable for neonates in high-altitude areas; this study aimed to establish birth growth curves for full-term neonates residing at 2,000–3,000 m.

**Methods:**

This cross-sectional study retrospectively analyzed the physical measurement data of 1,546 full-term neonates delivered at the Red Cross Hospital of Qinghai province, China, from July 2021 to April 2022. The percentile curves of birth weight, length, and head circumference of neonates of different gestational ages and genders were developed using curve fitting. The newly developed birth-weight percentile reference was compared with the INTERGROWTH-21st Neonatal Growth Curve (International Standard) and the Chinese Neonate Growth Curve (Chinese Standard).

**Results:**

The median birth weight, length, and head circumference of the study population were 3,200 g, 52.0 cm, and 32.8 cm, respectively, except for the group with a gestational age of 37 weeks. The growth indicators of male infants in all groups were higher than those of the female infants (*P* < 0.05). We found differences between the newly developed birth-weight percentile curves in the high-altitude areas and the International and Chinese Standards.

**Conclusion:**

Establishing birth growth curves corresponding to altitude may be more suitable than the existing standards for local medical staff to conduct health assessments of neonates.

## Introduction

Levels of physical development of newborns reflect their intrauterine growth and nutritional status, and they are also essential factors in the early survival and development after birth (1). Therefore, accurate health evaluations are critical. The birth growth curve is essential to evaluate whether the physical development of a child is abnormal. Its measurement includes the percentile curves of birth weight, length, and head circumference of infants of different gestational ages and genders (2).

Several widely used neonatal growth curves have emerged during the past decade. In 2013, Fenton et al. included data from developed countries, such as Italy, Germany, and the United States from 1991 to 2007 to develop the Fenton growth curve, which is widely used in several countries (3). In 2014, the INTERGROWTH-21st Project team released the neonatal growth curve (the international standard) based on data collected from medical centers in eight countries and individuals of various ethnicities (4). Despite some controversies about it (5, 6), the international standard is considered a good reference. In 2020, the Chinese National Health Commission implemented the Chinese neonatal growth curve (the Chinese standard) to enhance the health evaluations of the growth and development of Chinese neonates (7).

Since these widely used growth curves are based on data from populations at sea level or low altitudes, they are unsuitable for newborns at high altitudes. Recently, two meta-analyses showed that the average birth weight decreases by 54.7–96.98 g for every 1,000 m increase in height. In contrast, altitude has little or no effect on gestational age at birth (8, 9). Furthermore, multiple studies have reported a significant association between altitude and low birth weight, independent of economic status (10–12). Therefore, altitude should also be included as a variable of interest in the same analyses as gestational age and gender, and growth curves corresponding to altitude should be established.

Gonzales et al. (13) and Al-Shehri et al. (14) established neonatal growth curves at altitudes of 3,000–4,400 m in Peru and 3,133 m in Saudi Arabia, revealing that the curves established at high altitudes significantly differed from those at sea level or low altitudes. Residents of Qinghai province, China, located on the Qinghai-Tibet plateau, live at a wide range of altitudes. However, the altitude of approximately 80% of the population ranges from 2,000–3,000 m above sea level. Neonates have unestablished growth curves at altitudes of 2,000–3,000 m.

This study aimed to establish percentile curves for the birth weight, length, and head circumference of local full-term neonates of different gestational ages and genders in the Qinghai-Tibet plateau of China at an altitude of 2,000–3,000 m, to identify neonates with abnormal physical development more accurately.

## Materials and methods

### Study design

The physical measurement data of neonates delivered at Qinghai Red Cross Hospital in Xining City, Qinghai province, China, on the Qinghai-Tibet plateau were retrospectively analyzed in this cross-sectional study. The hospital is the largest tertiary delivery center in Qinghai province, with an altitude of 2,261 m above sea level. It serves pregnant women from different areas of Qinghai province to give birth. The Medical Ethics Committee of Qinghai Red Cross Hospital approved the study protocol (Approval number: LW-2022-27).

### Inclusion and exclusion criteria

In June 2021, quality improvement and training on taking physical measurements of neonates were implemented by the nursing department of center. After training, the measurements were checked and found to be reliable. The inclusion criteria for this study were: (1) all live neonates delivered at the Qinghai Red Cross Hospital between July 2021 and April 2022, and the altitude of mother residence was 2,000–3,000 m; (2) the neonatal gestational age at birth was within the range of 37^+0^–41^+6^ weeks. The exclusion criteria were: (1) incomplete information; (2) twin or multiple births; (3) artificial insemination of the mother; (4) birth with a congenital malformation, limb mutilation, fetal hydrops, or chromosomal abnormality; (5) mother age <18 years or >40 years; (6) the presence of any of the following diseases in the mother during pregnancy: severe anemia (hemoglobin ≤70 g/L), gestational diabetes mellitus, gestational hypertension, abnormal thyroid function, and heart or renal insufficiency, or (7) living in the stated residence <1 year. This information was obtained from the electronic medical record system of hospital.

### Data collection and definitions

Regarding neonates, the collected data included: gender, mode of delivery, gestational age, birth weight, length, and head circumference. Concerning mothers, the collected data included: age, gravidity, and parity. The involved study variables were as follows: (1) gestational age: the method of gestational age assessment of the center was based on the comprehensive determination of the last menstrual period and ultrasound examination results of the first trimester (first three months) of the mother. When a gestational age determined using the two methods differed by <one week, the gestational age at the time of the last menstrual period prevailed. When a gestational age determined by the two methods varied by ≥1 week, the gestational age determined by ultrasound prevailed. (2) Birth weight: was measured and recorded within 12 h of birth, and the reading was rounded to 10 g. (3) Length and head circumference: were measured and recorded within 24 h of birth, and the reading was rounded to 0.1 cm. (4) Residence: where the pregnant woman lived during her admission to the hospital for delivery. An altitude ≥2,000 m is a high altitude (15).

### Statistical analysis

This study was divided into groups by weeks of gestational age, with one group per each gestational week, the gestational age of the 37-week of gestation group was 37^+0^–37^+6^ weeks, and neonates in the 37–41 weeks of gestation group were divided into five groups with one group per week. Data collation, logistic checks, and basic statistical analysis were performed using SPSS v26.0 (IBM, Armonk, NY, USA). Data with a normal distribution were expressed as mean ± standard deviation (SD), frequencies were expressed as numbers (%), and data with skewed distributions were expressed as interquartile ranges. Outliers (mean ± 5 SDs) were removed when a reference standard was established. The percentile curve fittings of birth weight, length, and head circumference were performed using the GAMLSS 4.3-1 software package in R v3.1.2 (R Foundation for Statistical Computing, Vienna, Austria). The newly developed birth weight percentile reference values were compared with the reference values of the International and Chinese Standards, and a comparison chart was drawn using Origin version 2017 (OriginLab, Northampton, Ma, USA). All tests were two-tailed, and *P* < 0.05 was set as the study level of statistical significance.

## Results

### Baseline characteristics

Herein, 1,546 neonates with a gestational age of 37^+0^–41^+6^ weeks were included. Supplementary Figure S1 shows the participant selection flow diagram. The median age of mothers was 29 years, and the mean altitude of their residences was 2,391.9 m. Males represented 51.0% of the included neonates, and their median gestational age at birth was approximately 39 weeks. The median birth weight, length, and head circumference of the study population were 3,200 g, 52.0 cm, and 32.8 cm. Table 1 summarizes the general characteristics of the study population.

**Table 1:** General characteristics of the study population.

Characteristic	
Mothers’ Information:	
Age (years)	29 (26, 32)
Gravidity	2.00 (1.00, 3.00)
Altitude of residence (*m*)	2391.90 ± 254.73
Neonates’ Information:	
Gender	
Male	789 (51.0%)
Female	757 (49.0%)
Cesarean section (%)	640 (41.4%)
Gestational age (*w*)	39.43 (38.86, 40.14)
Birth weight (*g*)	3,200 (2928, 3440)
Length (cm)	52.0 (50.0, 52.0)
Head circumference (cm)	32.8 (32.0, 34.0)

Supplementary Table S1 summarizes the distribution ranges of the birth weight, length, and head circumference of neonates at gestational age 37^+0^–41^+6^ weeks. Table 2 summarizes the distribution ranges of growth indicators of male and female neonates. Except for the the group with a gestational age of 37 weeks, the growth of male infants was higher than that of the female infants in each gestational age group, and the differences were statistically significant (*P* < 0.05) (Table 2).

**Table 2:** Distribution range of growth and development indicators by gender and gestational age.

Gestational age (*w*)	Birth weight (*g*)		*P* value	Length (cm)		*P* value	Head circumference (cm)		*P* value
	Male	Female		Male	Female		Male	Female	
37	2,890 (2,538, 3,050)	2,870 (2,643, 3,115)	0.72	49.0 (47.0, 51.0)	50.0 (48.0, 51.0)	0.455	31.0 (29.0, 32.0)	32.0 (30.0, 32.0)	0.412
38	3,120 (2,920, 3,310)	2,925 (2,720, 3,233)	0.001	51.0 (50.0, 52.0)	50.0 (48.0, 52.0)	0.001	33.0 (31.0, 34.0)	32.0 (30.0, 33.0)	<0.001
39	3,260 (3,048, 3,503)	3,150 (2,920, 3,370)	0.001	52.0 (50.0, 52.3)	51.0 (49.0, 52.0)	0.007	33.0 (32.0, 34.0)	32.0 (31.5, 33.0)	0.048
40	3,340 (3,100, 3,630)	3,285 (3,005, 3,490)	0.004	52.0 (50.5, 53.0)	52.0 (50.0, 52.0)	0.002	33.0 (32.0, 34.0)	33.0 (32.0, 34.0)	0.035
41	3,525 (3,343, 3,700)	3,270 (2,893, 3,588)	0.04	53.0 (52.0, 53.0)	52.0 (49.0, 53.0)	0.006	33.5 (33.0, 34.8)	32.0 (31.3, 34.0)	0.012

### Developing birth growth curves

**Figure 1:**
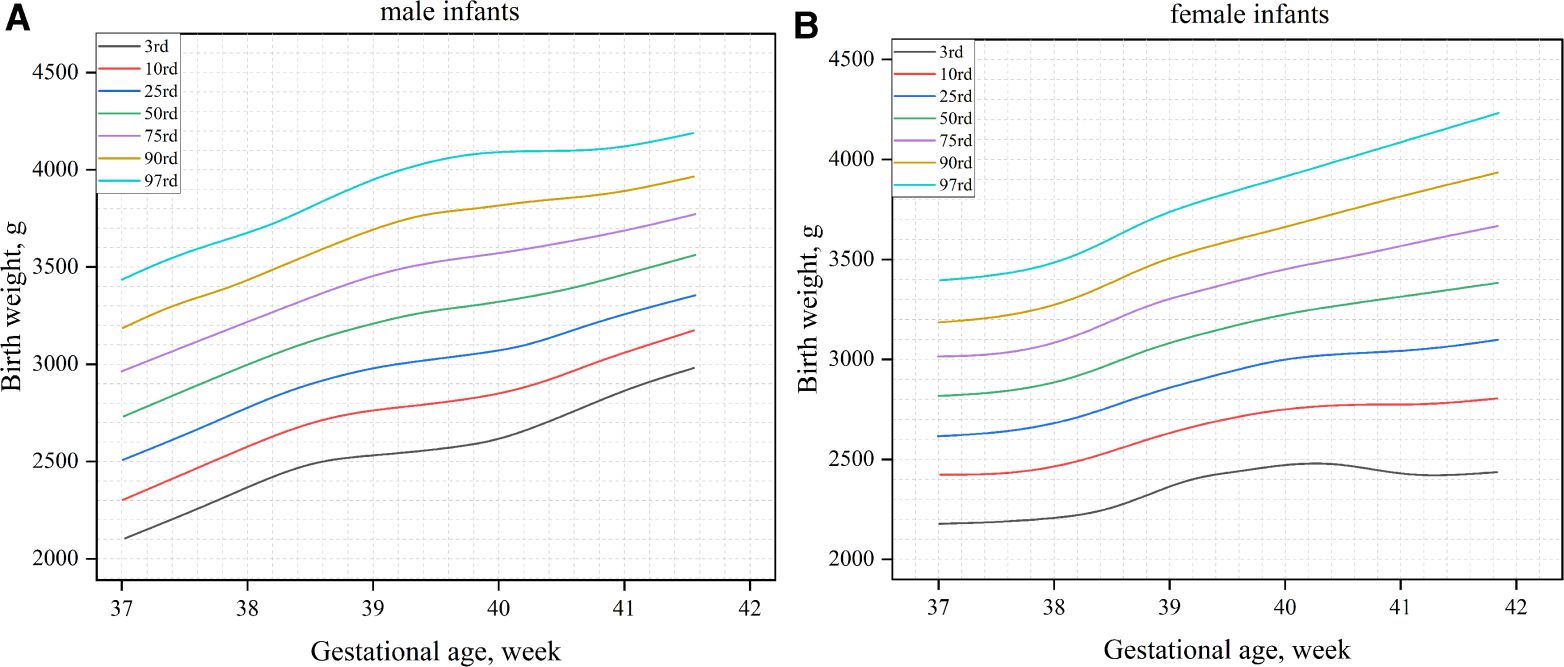
Birth weight percentile curves by gender and gestational age (**A**): male infants; (**B**): female infants.

**Figure 2:**
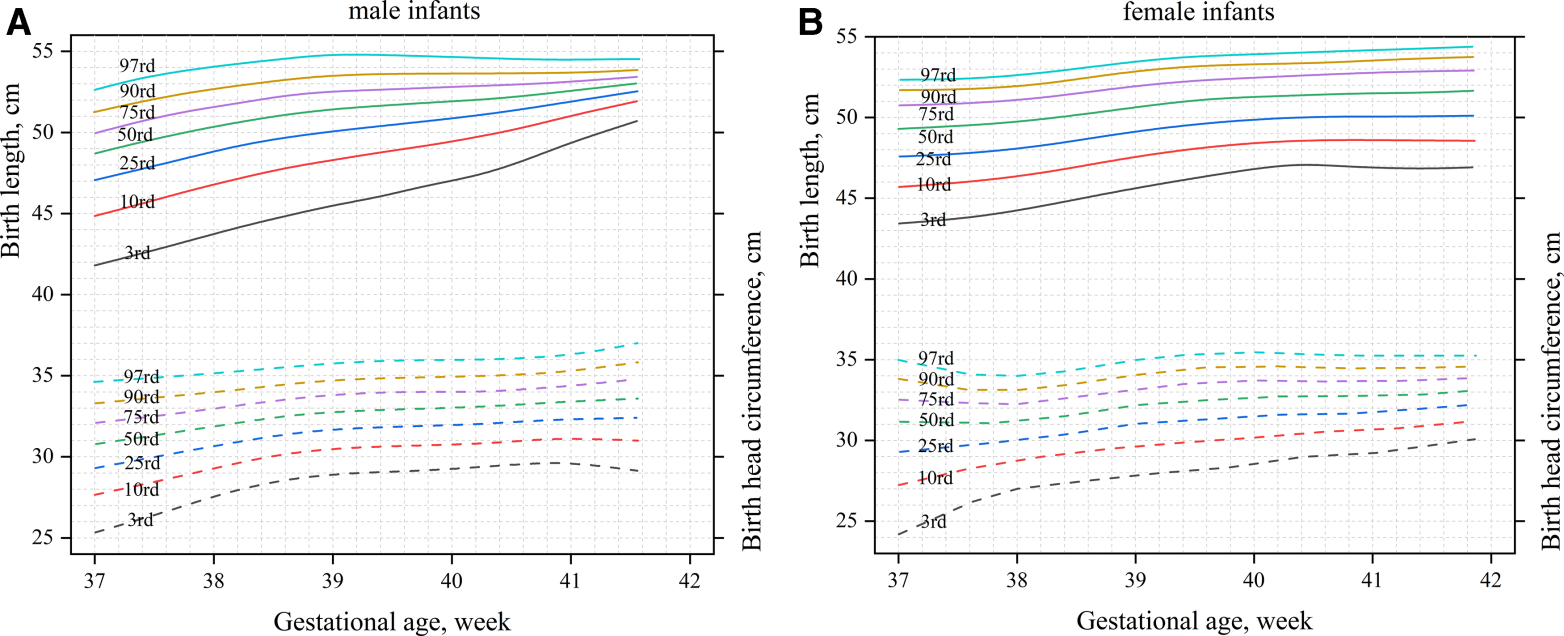
Percentile curves of the length and head circumference by gender and gestational age (**A**): male infants; (**B**): female infants.

Figure 1 shows the birth weight percentiles of neonates of different gestational ages and genders obtained using curve fitting. Figure 1A shows male infants, and Figure 1B shows female infants. Figure 2 shows the percentile curves of the length and head circumference of neonates. Figure 2A shows male infants, and Figure 2B shows female infants.

### Comparison between different standards

**Figure 3:**
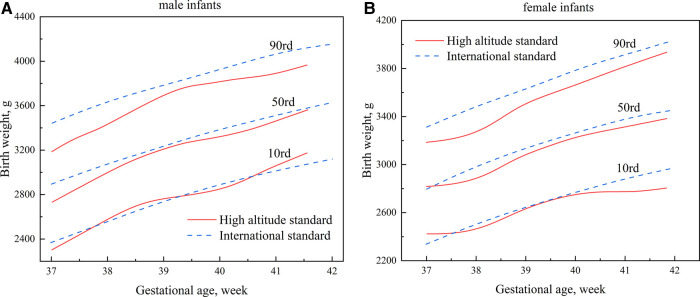
Comparisons of percentile curves of the high-altitude and international standards for neonatal birth weight (**A**): male infants; (**B**): female infants.

The newly developed percentile curves for the birth weight of newborns in high-altitude areas (the high-altitude standard) were compared with the international standard (Figure 3). On the 10th percentile (P10) curve of the birth weight of male neonates, the two Standards were relatively closer between 37 and 41 weeks (Figure 3A). On the 90th percentile (P90) curve of birth weight of male infants, the high-altitude standard was significantly lower than the international standard, and the maximum difference was approximately 252 g (Figure 3A). The two standards were relatively close between 39 and 40 weeks on the 10th percentile curve of the birth weight of female infants, with the high-altitude standard significantly lower than the international standard after 40 weeks. The maximum difference was about 157 g (Figure 3B). Similarly, to male infants, the high-altitude standard of female infants was significantly lower than the international standard on the 90th percentile curve of the birth weight of female infants, with a maximum difference of about 211 g (Figure 3B).

**Figure 4:**
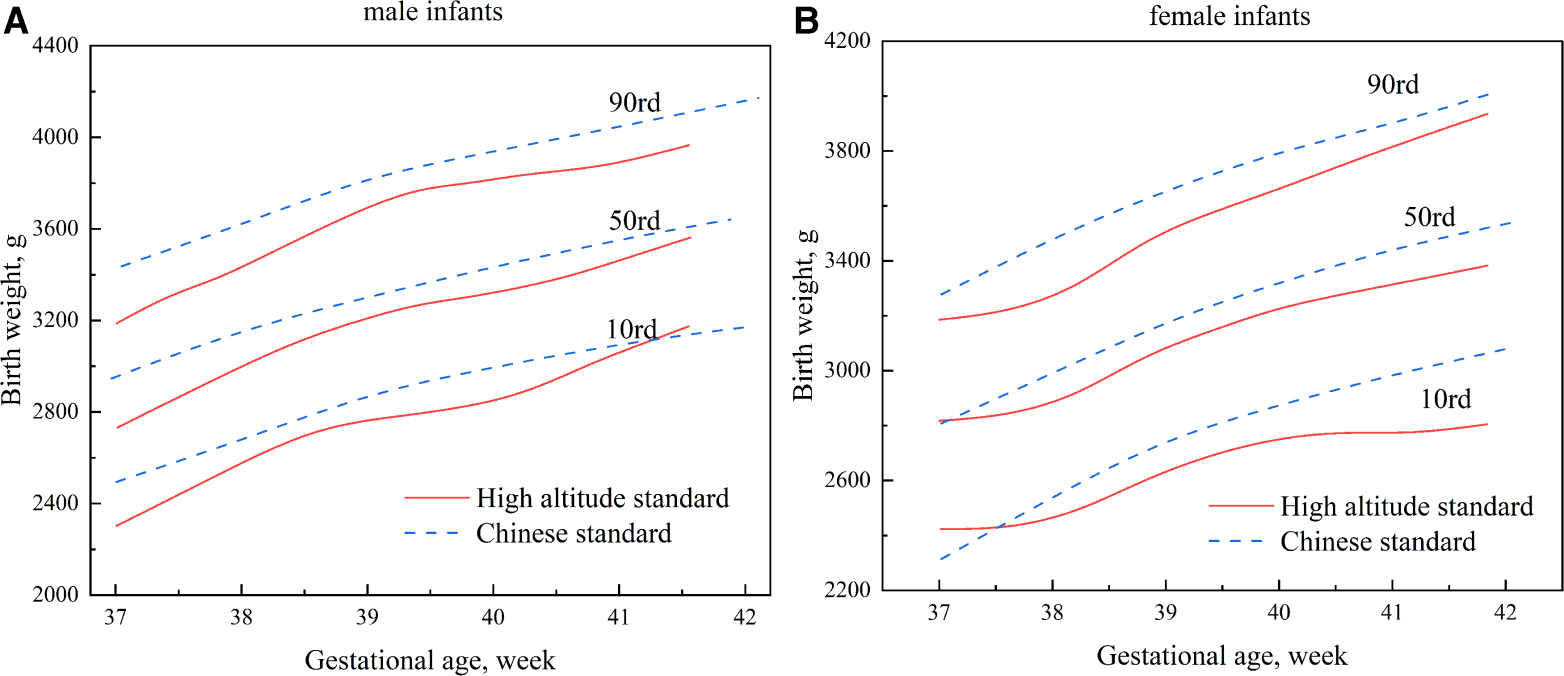
Comparisons of percentile curves of the high-altitude and Chinese standards for neonatal birth weight (**A**): male infants; (**B**): female infants.

Figure 4 compares the high-altitude standard and the Chinese Standard results. The two standards were relatively close at 41 weeks of gestational age on the 10th percentile curve of the birth weight of male infants; the remaining curves were lower than the Chinese standard, with a maximum difference of approximately 194 g (Figure 4A). Beginning at 37 weeks, the growth curve of the birth weight of female infants in the high-altitude standard was slightly higher than the Chinese standard. However, it gradually shifted downward and was lower than the Chinese standard, with a maximum difference of about 261 g (Figure 4B). On the 90th percentile curve, the high-altitude standard was significantly lower than the Chinese standard for male and female infants, with maximum differences of approximately 257 g and 203 g, respectively (Figure 4).

## Discussion

There are differences in the physical development of newborns between different populations and races, and it is not appropriate to use a one-size-fits-all approach, i.e., the same standard for all (16). The International Federation of Gynecology and Obstetrics also supports using regionally developed growth charts and considers them the best option for identifying high-risk neonates (17). Therefore, this study established birth growth curves for full-term neonates living at an altitude of 2,000–3,000 m in the Qinghai-Tibet plateau region of China. We found differences between the newly developed high-altitude and international and Chinese standards.

Altitude is an independent influencing factor significantly associated with low birth weight (8–12). Unfortunately, the physiological mechanisms are still incompletely understood. Low-pressure hypoxia at high altitudes may be the ultimate cause of fetal growth restriction (18, 19). The body develops compensatory erythropoiesis at high altitudes due to persistent chronic hypoxia to improve oxygen-carrying capacity and compensate for insufficient oxygen supply. However, excessive erythrocytes increase the viscosity of whole blood, slow blood flow, and increase the resistance to blood flow, which can cause insufficient perfusion of placental tissue. Furthermore, chronic hypoxia decreases pregnancy-related uterine artery blood flow, reducing placental blood perfusion and affecting fetal growth and development. Finally, the placenta may respond directly to changes in blood oxygen concentration or maternal physiology (blood viscosity or pressure), resulting in structural remodeling of the placenta, with thickening of the trophoblastic basement membrane and an increase in collagen fibers. This phenomenon can squeeze the blood vessels and obstruct the normal perfusion of villi, resulting in impaired exchange of air, blood, and substances between mother and child, affecting development.

There are theoretical differences in neonatal physical development between 2,000 m and 3,000 m altitude. However, we considered 2,000 m–3,000 m as a whole for several reasons. First, creating separate growth curves for each altitude region is impractical. Second, the Chinese Qinghai province is located on the Tibetan plateau, and the population lives in a wide range of altitude distributions, and the same city may have multiple altitudes. Developing reference standards needs to consider their practicality and convenience fully. About 80% of the population of Qinghai province in China lives in the range of 2,000 m–3,000 m above sea level. Third, by comparison, the high-altitude standard we made is better than the current standard, although it still has a gap with the theory. Fourth, this practice is commonly used now. For example, Gonzales GF et al. (13) considered the altitude of 3,000 m–4,400 m as a whole and created the growth curve of neonates in this area.

This study treated the residence altitude of mother during her pregnancy as an involved variable. However, many previous studies on the effects of altitude on the physical development of neonates have examined the birthplace altitude as a study variable (13, 14, 20). This practice is inappropriate because the gap between the residence altitude of the mother and the birthplace of child could affect the study results. This study also excluded potential participants with complications during pregnancy (gestational diabetes) because Hoftiezer et al. (21) found that complications during pregnancy, such as gestational hypertension and diabetes, affect the neonatal birth size. Therefore, risk factors that may lead to abnormal fetal growth, including maternal comorbidities during pregnancy, should be excluded when establishing a birth growth curve (7). Villamonte-Calanche et al. (22, 23) found a neonatal growth curve at 3,400 m in Peru, similar to that of the international standard, unlike our results. However, their study ignored factors, including residence altitude or pregnancy comorbidities. The international standard has strict altitude limits and it excludes high-risk groups during pregnancy, which may account for the different results from our study.

Lubchenco et al. (24) found that the neonatal mortality rate increased significantly when the birth weight was below (P10) of the reference curve. They used the P10 and P90 of the birth weight reference curve as cut-off points to distinguish neonates as small for gestational age (SGA), appropriate for gestational age (AGA), or large for gestational age (LGA). This classification is still used today. This study established a P10 curve of the high-altitude standard, which was relatively close to the international standard, but the P90 was significantly lower than the international standard. If the international standard were used as our reference standard, some LGA neonates would inevitably be overlooked. Compared to the Chinese standard, which is more suitable for the Chinese population, the P10 and P90 of the high-altitude standard were almost lower than the Chinese standard for male and female infants. This leads to an underestimation of LGA neonates and an overestimation of SGA neonates. This is unfavorable in high-altitude areas where the economy is modest and medical resources are scarce. It highlights the importance of establishing a growth curve suitable for high altitudes. Accordingly, it is worth investigating the suitability of using the P10 and P90 as cut-off points for screening high-risk neonates in high-altitude areas.

The level of physical development of neonates at birth is a significant public health indicator that reflects fetal health and predicts future health (25). Our results will provide a theoretical basis for formulating public health policies in high-altitude areas. The declining economy, poor living conditions, and low medical resources in high-altitude areas make it essential to accurately assess the physical development of neonates at birth to allocate limited medical resources to babies who need help efficiently.

This study has some limitations. First, the relatively small number of neonates in the 37- and 41-week gestational age groups, 123 (63 for male infants) and 48 (20 for male infants), respectively. However, methods to calculate sample size for establishing growth standards have yet to be fully developed (26). There is no uniform standard, and the international standard requires that the total sample size of each country be at least 7,000. However, no specific requirements have been established for the sample size of different gestational age groups or gender groups. The smallest sample size of other groups included in the INTERGROWTH-21st Project study was only 17 (4). The Chinese standard was formulated following the statistical requirements of the WHO Special Survey on Physical Development (27). The sample size of males and females in each gestational age group of full-term neonates was ≥100, while that of the preterm neonates was ≥50. No sample size has been evaluated or recommended for extreme gestational age groups, including those <28 weeks (7, 28). The final minimum sample size of different groups was 15 participants. The small population at high altitudes makes sample collection difficult. As the largest delivery center in Qinghai province, the sample size collected by this center is challenging for high altitudes in China. Second, the data was extracted from the electronic medical record system of hospital. However, data on risk factors, including the height of mother, may affect the fetal size and failed to be extracted due to system flaws. Finally, since obtaining the original data for producing Chinese and international standards is impossible, this study used a direct comparison method rather than statistical methods. To address these limitations, we have planned a multicenter study with a rigorous prospective design to develop birth growth curves for neonates at high altitudes, including preterm neonates.

## Conclusions

It is essential to base the selection of growth curves on altitude to assess the physical development of local neonates. This study was the first to develop birth growth curves for different genders of full-term neonates residing at an altitude of 2,000–3,000 m. Differences were observed between the newly developed birth-weight percentile curves in the high-altitude areas and the International and Chinese Standards. Meanwhile, it is necessary to further explore the birth growth curves of neonates at high altitudes, including preterm neonates.

## Data Availability

The raw data supporting the conclusions of this article will be made available by the authors, without undue reservation.
