# Supplementary material for: Birth growth curves of neonates in high-altitude areas: A cross-sectional study
Source: Front Pediatr. 2023 Jan 10;10:1028637. doi: 10.3389/fped.2022.1028637 (PMC9871478; doi:10.3389/fped.2022.1028637)
Supplement: Supplementary file 1 [file Table1.docx]

**Supplementary Table 1.** Distribution range of growth and development indicators by gestational age

| Gestational age (w) | N | Male | Birth weight (g) | Length (cm) | Head circumference (cm) |
| --- | --- | --- | --- | --- | --- |
| 37 | 123 | 67(54.5%) | 2870（2600，3080） | 49.0（48.0，51.0） | 32.0（30.0，32.0） |
| 38 | 289 | 159(55.0%) | 3030（2800，3280） | 50.0（49.0，52.0） | 32.0（30.0，33.0） |
| 39 | 631 | 322(51.0%) | 3220（2980，3440） | 52.0（50.0，52.0） | 33.0（32.0，34.0） |
| 40 | 455 | 221(48.6%) | 3300（3050，3580） | 52.0（50.0，53.0） | 33.0（32.0，34.0） |
| 41 | 48 | 20(41.7%) | 3395（2978，3608） | 52.0（50.0，53.0） | 33.0（32.0，34.0） |
