# Supplementary figures and images for: Birth growth curves of neonates in high-altitude areas: A cross-sectional study
Source: Front Pediatr. 2023 Jan 10;10:1028637. doi: 10.3389/fped.2022.1028637 (PMC9871478; doi:10.3389/fped.2022.1028637)

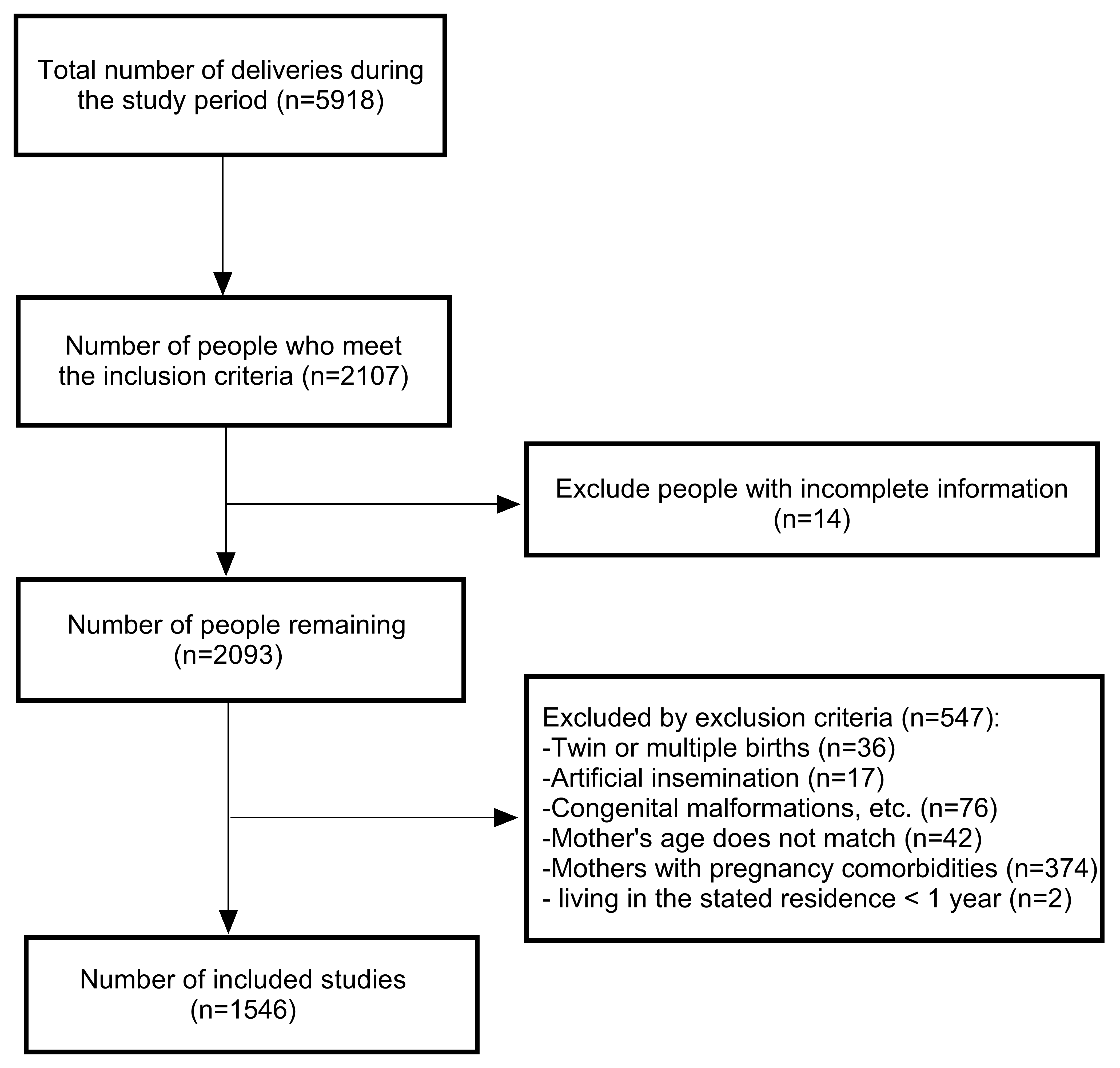

Supplement: Supplementary file 2 [file Image1.tif]
